# At the Interface of Life and Death: Post-mortem and Other Applications of Vaginal, Skin, and Salivary Microbiome Analysis in Forensics

**DOI:** 10.3389/fmicb.2021.694447

**Published:** 2021-07-28

**Authors:** Sarah Ahannach, Irina Spacova, Ronny Decorte, Els Jehaes, Sarah Lebeer

**Affiliations:** ^1^Department of Bioscience Engineering, Research Group Environmental Ecology and Applied Microbiology, University of Antwerp, Antwerp, Belgium; ^2^Laboratory of Forensic Genetics, Department of Forensic Medicine, University Hospitals Leuven, Leuven, Belgium; ^3^Department of Imaging and Pathology, Forensic Biomedical Sciences, KU Leuven, Leuven, Belgium; ^4^Forensic DNA Laboratory, Department of Forensic Medicine, Antwerp University Hospital, Edegem, Belgium

**Keywords:** post-mortem microbiome, trace evidence, microbial forensics, sexual assault, femicide, next-generation sequencing, thanatomicrobiome, epinecrotic communities

## Abstract

Microbial forensics represents a promising tool to strengthen traditional forensic investigative methods and fill related knowledge gaps. Large-scale microbiome studies indicate that microbial fingerprinting can assist forensics in areas such as trace evidence, source tracking, geolocation, and circumstances of death. Nevertheless, the majority of forensic microbiome studies focus on soil and internal organ samples, whereas the microbiome of skin, mouth, and especially vaginal samples that are routinely collected in sexual assault and femicide cases remain underexplored. This review discusses the current and emerging insights into vaginal, skin, and salivary microbiome-modulating factors during life (e.g., lifestyle and health status) and after death (e.g., environmental influences and post-mortem interval) based on next-generation sequencing. We specifically highlight the key aspects of female reproductive tract, skin, and mouth microbiome samples relevant in forensics. To fill the current knowledge gaps, future research should focus on the degree to which the post-mortem succession rate and profiles of vaginal, skin, and saliva microbiota are sensitive to abiotic and biotic factors, presence or absence of oxygen and other gases, and the nutrient richness of the environment. Application of this microbiome-related knowledge could provide valuable complementary data to strengthen forensic cases, for example, to shed light on the circumstances surrounding death with (post-mortem) microbial fingerprinting. Overall, this review synthesizes the present knowledge and aims to provide a framework to adequately comprehend the hurdles and potential application of vaginal, skin, and salivary post-mortem microbiomes in forensic investigations.

## Introduction

Violence against women is an urgent global problem, as more than one-third of women worldwide has been victim of physical and/or sexual violence in their lifetime (Devries et al., 2013; World Health Organization, 2013). Annually, 66,000 women and girls are victims of femicide, that is, intentional murder of women and girls because they are female (Geneva Declaration, 2011; World Health Organization and Pan American Health Organization, 2012). Remarkably, only 25% of reported rape cases in Europe lead to a conviction, often due to the difficulty of providing evidence (Lovett and Kelly, 2009). Moreover, forensic experts are convinced that a large number of undetected homicides are misclassified in annual death statistics as natural deaths, suicides, or accidents (Karger et al., 2004; Ferguson and McKinley, 2019). The major obstacle is the difficulty of elucidating the circumstances surrounding death, including cause and manner (Pechal et al., 2018; Kaszubinski et al., 2020b). Traditional forensic techniques, such as human DNA profiling, can provide critical evidence by linking biological traces to crime scenes and individuals or through victim identification (Franzosa et al., 2015). However, they occasionally fall short because of human DNA degradation (Sijen, 2015; Ranjan and Surajit, 2018) and need to be complemented with alternative techniques.

Recent advances in microbial profiling have uncovered that each individual is home to complex microbial communities (Oh et al., 2016; Gilbert and Stephens, 2018). These communities inhabit all surfaces of the human body (for example, orogastrointestinal tract, respiratory tract, urogenital tract, skin) and collectively represent the human microbiota, with their microbial DNA signatures forming the microbiome (Ursell et al., 2012; Berg et al., 2020). Recent research suggests that the microbiome could greatly aid forensic casework (Clarke et al., 2017; Hampton-Marcell et al., 2017; Metcalf et al., 2017; Oliveira and Amorim, 2018; Bishop, 2019). For example, the microbiome can serve as a personal microbial fingerprint that not only can associate individuals to objects (Lax et al., 2015) and geographical locations that they came in contact with (Knight et al., 2016), but also can provide identifiable characteristics (Franzosa et al., 2015). Moreover, many microbial cells contain robust cell walls that leave them better protected against degradation compared to human cells (Sijen, 2015). Nevertheless, the focus of microbial forensics to date has been predominantly on gut and soil samples (Metcalf et al., 2013, Metcalf et al., 2016; Pechal et al., 2014; Javan et al., 2016b; Burcham et al., 2019; DeBruyn et al., 2021), whereas research on forensic implementation of the (post-mortem) microbiome of the female reproductive tract, skin, and oral cavity is lagging behind. Therefore, in this review, we provide a critical assessment of current research on the vaginal, skin, and oral/salivary microbiome in relation to their potential application in forensics, especially sexual assault and femicide cases.

## Relevance of Vaginal, Skin, and Salivary Microbiome During Life for Forensic Casework

Vaginal, skin, and saliva samples represent some of the most commonly collected samples in forensic casework, including sexual assault cases (World Health Organization, 2003; Mont and White, 2007) and cases involving touch evidence (Burrill et al., 2019; Oorschot et al., 2019). These mucocutaneous niches are shaped by several microbiome-influencing factors (e.g., pH and oxygen) (Rojo et al., 2017; Burcham et al., 2019). However, which of these factors have the largest effect is still unknown.

**FIGURE 1:**
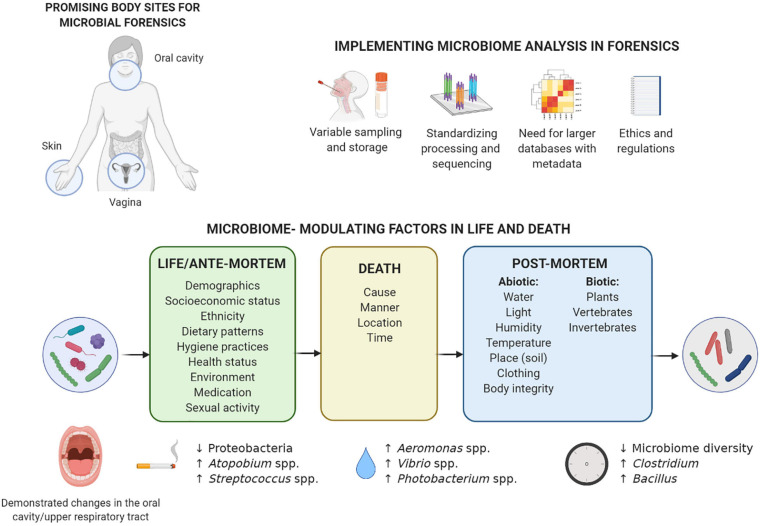
Considerations for implementation of microbiome analysis in forensics and overview of factors in life and death that can influence the microbiome composition. The microbiome of the three body sites understudied in forensics (vagina, skin, and oral cavity) can be influenced by various individual factors during life, and these influences can sometimes also be detected after death. Death forms a turning point for the microbiome: the post-mortem microbiome is much more influenced by a range of environmental factors. Previously described microbiome-modulating factors in life and death are summarized, with some of the most prominent examples given of specific taxa in the oral cavity/upper respiratory tract associated with smoking during life (Wu et al., 2016), drowning as cause of death (Uchiyama et al., 2012; Benbow et al., 2015), and time that has passed since death (Adserias-Garriga et al., 2017b; Javan et al., 2017; García et al., 2020). More examples for the other body sites can be found in the text, although, to the best of our knowledge, no data are available on the post-mortem microbiome of the vagina. Implementation of microbiome analysis in forensics still requires extensive standardization of sampling and processing, as well as larger reference databases with metadata and an adjusted regulatory and ethical framework. Created with BioRender.com.

Vaginal samples are routinely collected in sexual assault cases (Quaak et al., 2018; Ghemrawi et al., 2021). While their microbiome is generally neglected in forensics, the less diverse composition of vaginal microbiota, its high microbial biomass, and protected anatomical location translate into its unique potential for microbial fingerprinting (Younes et al., 2018). Depending on the women’s ethnicity, the vaginal microbiome is generally dominated by Gram-positive *Lactobacillus* genera covered by a thick cell wall (i.e., *Lactobacillus crispatus*, *Lactobacillus iners*, *Lactobacillus gasseri*, and *Lactobacillus jensenii*) or a diverse microbiota dominated by non-lactobacilli such as *Bifidobacterium*, *Gardnerella*, *Atopobium*, and *Prevotella* (Ravel et al., 2010). Also fungal taxa mostly represented by *Candida* are detected, but generally in low abundances in healthy women (Chew et al., 2016). Ongoing research suggests that the vaginal microbiome composition can be correlated to individual characteristics valuable in forensics, such as health status (Ceccarani et al., 2019), ethnicity (Borgdorff et al., 2017; Gupta et al., 2017), sexual habits (Noyes et al., 2018), contraceptive use (Song et al., 2020), and pregnancy (Serrano et al., 2019), with various effect sizes that are not yet well mapped (Figure 1). For example, a longitudinal study found that sexual activity within 24 h of sampling has a significant negative impact on vaginal microbiome constancy as measured *via* the log Jensen–Shannon divergence rate (i.e., vaginal community deviation from constancy), independent of time in the menstrual cycle (Gajer et al., 2012). The vaginal microbiome could thus represent trace evidence in sexual assault cases indicating sexual intercourse in the last 24 h, in addition to providing links with other identifiable individual characteristics. However, whether this conclusion can be drawn from single, non-longitudinal samples after sexual intercourse needs to be investigated.

While vaginal samples are especially useful in sexual assault cases, the skin is probably the most commonly used source of forensic trace evidence, including skin under the victim’s fingernails (Metcalf et al., 2017; Burrill et al., 2019). However, the unique microbial trail left behind by skin shedding (Bishop, 2019; Hampton-Marcell et al., 2020) is often overlooked. The skin microbiome is dominated by Gram-positive *Staphylococcus*, *Corynebacterium*, *Cutibacterium*, *Streptococcus*, or *Micrococcus*, although Gram-negative *Acinetobacter* are also frequently isolated (Grice and Segre, 2011). The skin microbiome composition varies depending on the body location (Costello et al., 2009; Grice and Segre, 2011), host characteristics (e.g., age, lifestyle, and cohabitation) (Ross et al., 2017), and skin care (Bouslimani et al., 2019). Importantly, the skin microbiome is at an interface between the outside world and the body that undergoes most interactions with the environment. In fact, detectable amounts of skin microbiota can be transferred to objects such as a computer keyboard and mouse (Fierer et al., 2010), shoes and phones (Lax et al., 2015), door handle, toilet seat, etc. (Flores et al., 2011). Remarkably, this is not only limited to touched surfaces, but also extends to inhabited spaces through microbial clouds which are detectable within just a few hours (Lax et al., 2014; Meadow et al., 2015). However, whether built environment microbiota can be used as trace evidence remains to be substantiated (Hampton-Marcell et al., 2020). An important knowledge gap is to what extent the skin microbiome can be detected on touched objects after a certain amount of time, potentially even after death. Establishing this link is complicated, as it depends on environmental parameters (e.g., temperature, moisture, and UV radiation) (Fierer et al., 2010), individual shedder status (i.e., amount of epithelium deposited on a substrate) (Lowe et al., 2002; Kanokwongnuwut et al., 2018), and surface characteristics of the object (Meadow et al., 2014).

Saliva, as the primary oral cavity sample, is another widely used trace in forensic casework, especially for skin bite marks in sexual assault and child abuse cases (Chávez-Briones et al., 2015; Leake et al., 2016). The salivary microbiome is mostly dominated by the Gram-negative *Neisseria, Prevotella*, or *Veillonella* but also contains large proportions of Gram-positive *Streptococcus* taxa (e.g., *Streptococcus salivarius* and *Streptococcus oralis*) (Willis et al., 2018). Oral Gram-positive bacteria have recently been described as robust markers for highly degraded saliva samples, because of their higher resistance to degradation treatment (e.g., heat denaturation, microbial decomposition, and ultraviolet irradiation) compared to Gram-negative salivary bacteria, salivary α-amylase, and human DNA (Ohta and Sakurada, 2019). Individual characteristics that can influence the salivary microbiome composition include smoking (Belstrøm et al., 2014; Wu et al., 2016), dental hygiene (Mashima et al., 2017; Burcham et al., 2020), general and oral health (Zhou et al., 2016; Goodson et al., 2017), and socioeconomic status (Belstrøm et al., 2014; Figure 1). Importantly, shared environment at household level appears to more significantly determine the salivary microbiome than individual genetics (Shaw et al., 2017). Also intimate contact relevant in forensics, such as kissing (i.e., mixing of saliva), has been proposed to impact salivary microbial composition. Specifically, a transfer of approximately 80 million marker bacteria per intimate kiss of 10 s is observed, and partners have a more similar microbial community compared to unrelated individuals (Kort et al., 2014). Further research into factors influencing the vaginal, skin, and oral/salivary microbiomes during life will allow their more targeted implementation in forensic casework.

## The Detrimental Effects of Death on the Human Microbiome

While the human microbiome during life is widely studied, we are just beginning to understand the post-mortem microbial community dynamics and how it can be influenced by ante-mortem microbial communities and modulating factors. During different stages after death, many anatomical and immunological barriers break, causing fluids, chemicals, and microorganisms that normally would not interact, to come into contact with each other (Gunn and Pitt, 2012). The post-mortem process facilitates the proliferation and relocation of microorganisms throughout the body and opens a gateway of cross-kingdom ecological interactions (Goff, 2009; García et al., 2020).

The decomposition of a human body is a continuous process caused by enzymatic reactions, (bio)chemical metabolic pathways and the activity of vertebrates and invertebrates (Pechal et al., 2018). This process is divided into a series of observable stages: fresh, active decay (including bloating and leakage of effusion), advanced decay, and dry remains/skeletonized (Goff, 2009; García et al., 2020). The course of these stages is partially determined by the diverse microbial communities occupying various internal and external body sites (Hauther et al., 2015; Javan et al., 2016a). Most studies on the post-mortem microbiome focus on estimating the minimum period of time since death [i.e., post-mortem interval (PMI)] (Goff, 2009). These studies aim to predict changes in the microbial composition of internal organs (e.g., gut, brain, liver, spleen, heart, etc.), also referred to as the post-mortem microbial clock (Metcalf et al., 2013; Finley et al., 2015).

Interestingly, studies on internal organs of mice (Metcalf et al., 2013; Burcham et al., 2019), swine (Carter et al., 2015), and human bodies (Hyde et al., 2013; Can et al., 2014; Hauther et al., 2015) have observed a shift in microbial communities from predominant aerobic microorganisms such as *Staphylococcus* and Enterobacteriaceae to more facultative anaerobic bacteria such as Proteobacteria, Firmicutes, and Bacteroidetes to obligate anaerobic organisms such as *Clostridium*, and finally spore-forming microorganisms such as *Clostridium* and *Bacillus* (Figure 1; Hyde et al., 2015; Javan et al., 2016b, 2019; García et al., 2020). According to the “post-mortem *Clostridium* effect,” *Clostridium* species can be considered important drivers of this microbial shift due to their lipolytic enzymes (Janaway et al., 2009), proteolytic functions, and rapid generation time (Javan et al., 2017). However, whether the findings from studies focusing on internal organs can be extended to mucocutaneous niches entails a different narrative.

Surprisingly, to date, no post-mortem microbiome studies have examined the vaginal microbiome succession after death (Table 1). This can be explained by the limited population of decomposing human bodies (mostly white males; >65 years old) studied at anthropological research facilities (Pechal et al., 2018). Nevertheless, Lutz et al. (2020) found that reproductive organs (i.e., uterus and prostate) were the last internal organs to decay. Particularly, for the nulligravid uterus (i.e., never been pregnant), the post-mortem *Clostridium* effect was not observed in contrast to the prostate and other internal organs. Of note, during life, the uterine microbiome is distinct from the vagina with a significantly lower microbial biomass and colonization by Firmicutes, Bacteroidetes, Proteobacteria, and Actinobacteria (Garcia-grau et al., 2019), and it is not clear to what degree bacterial transfer from the vagina to the uterus occurs after death. This highlights the underexplored potential of the female reproductive tract in post-mortem research.

**TABLE 1:** List of human post-mortem microbiome studies which include female reproductive tract, skin and/or oral cavity samples in the last 5 years.

**Niches**	**Study aim**	**Sequencing**	**Population and sample size**	**Main outcome**	**Main pitfall**	**References**
Brain, heart, liver, spleen, prostate, and **uterus**	Estimating minimum PMI and cause of death	16S rRNA gene amplicon sequencing	158 samples	Reproductive organs (uterus and prostate) were the last internal organs to decay during human decomposition	Larger population size is needed to further account for variation due to (a)biotic factors	Lutz et al., 2020
			40 human bodies (14 female, 26 male)			
			6 body sites			

**Skin:** nose and ear	Estimating minimum PMI	16S rRNA gene amplicon sequencing	144 samples	Machine learning model predicted the PMI with an average error of 2 days	Model was based on only four human bodies that were sampled longitudinally	Johnson et al., 2016
			21 human bodies			
			2 body sites			

**Skin:** left hip, right hip, left bicep, right bicep, left upper hip, right upper hip, left knee, groin, head	Understanding microbially mediated processes during decomposition on different soil substrates	16S rRNA gene amplicon sequencing 18S rRNA gene amplicon sequencing ITS amplicon sequencing	2 human bodies during winter	Soil type was not a dominant factor driving community development in the process of decomposition	Limited population size with no information on sex	Metcalf et al., 2016
			3 skin sites			
			143 days			
			2 human bodies during spring			
			8 skin sites			
			82 days			

Eyes, **ears**, **mouth,** nose, rectum, **thigh skin**	Estimating minimum PMI for buried bodies	16S rRNA gene amplicon sequencing	2 male bodies	Multidisciplinary methodology identified temporal changes in morphology, skeletal muscle protein decomposition, entomology, and microbiome for buried bodies	Model was based on only two human bodies of which multiple samples were taken	Pittner et al., 2020
			10 timepoints			

**Skin:** right hand palm	Linking objects at the death scene to deceased individuals	16S rRNA gene amplicon sequencing	11 male bodies	Objects could be traced to deceased individual 75% of the time	Ante-mortem population was not always a demographic representation of the deceased study population	Kodama et al., 2019
			5 female bodies			
			30 living individuals			
			79 skin samples			
			98 object samples			

Eyes, nose,**ears, mouth, umbilicus** rectum	1. Predicting the ante-mortem health condition of the deceased 2. Comparing three machine learning methods to predict PMI, location of death, and manner of death 3. Predicting cause and manner of death	16S rRNA gene amplicon sequencing	47 male bodies 141 female bodies 6 body sites 1 timepoint	1. Microbial biodiversity from the mouth could predict ante-mortem host health condition (e.g., heart disease) 2. Analysis of post-mortem microbiota from more than thee anatomic areas had limited additional value 3. Beta-dispersion, and case demographic data reflected forensic death determination	Only one timepoint (majority of cases with estimated PMI of <72 h) which does not account for variability within a body	1. Pechal et al., 2018 2. Zhang et al., 2019 3. Kaszubinski et al., 2020a

**Mouth:** palate, tongue, inner cheek mucosa and tooth surfaces	Estimating minimum PMI	16S rRNA gene amplicon sequencing	1 male body	Post-mortem microbial succession in the oral cavity changed in a temporal way according to oxygen availability	Limited population size with large variability	Adserias-Garriga et al., 2017b
			2 female bodies			
			8 timepoints			
			5 body sites			

**External auditory canal**, eyes, nares, **mouth**, **umbilicus**, and rectum	Studying the impact of coexisting conditions such as frozen affect the human microbiome at the time of discovery	16S rRNA gene amplicon sequencing	1 male body 1 female body 3 timepoints	The microbial diversity increased throughout the thawing process	Association with time since death or cause of death	Pechal et al., 2017

Blood, brain, **buccal cavity**, heart, liver, and spleen	Estimating minimum PMI	16S rRNA gene amplicon sequencing	66 samples	Microbial communities demonstrated time-, organ-, and sex-dependent changes	Niche sampling was not equal for all deceased individuals	Javan et al., 2016b
			27 human bodies (12 female, 15 male)			
			6 body sites			

**Mouth**, external left/right **cheeks** external left/right **bicep** region, **torso**, and rectum	Studying outdoor decomposition under natural conditions	16S rRNA gene amplicon sequencing and 454 pyro- sequencing	1 male body	Shifts in community structure were recorded and associated with major decomposition and related events	Limited population size with large variability	Hyde et al., 2015
			1 female body			
			10 timepoints			

*The bold terms refer to the most relevant niches.*

While current research has focused on the potential of the skin microbiome as trace evidence (Tozzo et al., 2020), to the best of our knowledge, only Kodama et al. (2019) have investigated whether actual objects from real death scenes (e.g., smoking pipes, medical devices, and phones) could be linked to the hand palm of the deceased through microbiome identification. The skin microbiome on the palm of the deceased remained stable up to 60 h after death, opening a window for individual microbiome identification even after death. It is noteworthy that this persistence of the skin microbiome into the early post-mortem period opens the possibility of also applying the post-mortem skin microbiome in PMI estimation. This is especially advantageous in cases where an autopsy is not requested, and a non-invasive microbiome sampling approach is best, because the most useful body sites for PMI estimation are external sites (e.g., skin).

Another body site easily accessible for microbiome and other sampling is the oral cavity. While its application for PMI estimation is yet to be studied in large populations, an increase of Firmicutes and Actinobacteria as the PMI increased was demonstrated (Hyde et al., 2013; Adserias-Garriga et al., 2017b). Interestingly, mouth samples pre-bloating resembled the oral microbiome during life, whereas the mouth samples post-bloating contained gut bacteria such as Tenericutes that possibly migrated from the large intestine (Adserias-Garriga et al., 2017b). Overall, studies that include more body sites, like Pechal et al. (2018) and others discussed in Table 1, could improve estimations.

The rate and pattern of decomposition are a mosaic system associated with biotic factors (e.g., individuality of the body, intrinsic and extrinsic bacteria, other microbes, and arthropods) and abiotic factors (e.g., weather, climate, humidity, and edaphic conditions) (Hyde et al., 2013; Carter et al., 2015; Newsome et al., 2021; Figure 1). It is yet to be elucidated how the contact of skin and natural body openings (mouth and vagina) with the outside environment (clothing, soil, aquatic ecosystems, etc.) can differentially influence the post-mortem body site-specific microbiome. For the latter, the application of epinecrotic communities such as aquatic microbes on the post-mortem submersion interval estimation could be highly relevant in aquatic death investigations (Benbow et al., 2015; Cartozzo et al., 2021; Randall et al., 2021). While the exact effect sizes are rarely reported (Meurs, 2016), abiotic factors, such as insects and soils beneath a decomposing body (Cobaugh et al., 2015; Metcalf et al., 2016; Adserias-Garriga et al., 2017a; Keenan et al., 2019), seasonal variation, and distinct climates (Carter et al., 2015), but also exposure and clothing (Goff, 2009), are some of the driving determinants of the microbial succession after death. Specifically, because of lack of thermoregulation, ambient temperatures (Goff, 2009) greatly affect the shift in nutrient availability and can thereby affect microbial community dynamics. Thus, while most studies have been performed in the United States (Metcalf et al., 2013; Pechal et al., 2018; DeBruyn et al., 2021) with a few in Australia, Japan, and the United Kingdom (García et al., 2020), post-mortem microbiome research in a wider range of climates should be encouraged.

## Potential Hurdles and Considerations

Juries in the court of law have come to rely on physical evidence to corroborate a testimony (Shelton et al., 2007). However, before microbiome research can be reliably introduced into investigative and legislative casework, it has to be peer-reviewed, standardized, and accepted by the scientific community (Kiely, 2005).

Microbiome sequencing methods can be divided into those targeting specific parts of microbial DNA, such as the widely used 16S rRNA gene amplicon sequencing, or untargeted approaches, such as metagenomic shotgun sequencing. In-depth shotgun metagenomics is relatively new and currently more expensive than 16s rRNA gene amplicon sequencing, but it offers the advantage of sequencing the whole genetic content (microbial and human) of a sample with a higher taxonomic and functional resolution (Quince et al., 2017; Schmedes et al., 2017; Hillmann et al., 2018; Walker and Datta, 2019). However, currently, amplicon sequencing is most widely implemented and thus relies on larger available datasets with metadata on microbiome-modulating factors necessary for increasing method accuracy of machine learning–based tools (Clarke et al., 2017; Belk et al., 2018; Zhang et al., 2019), allowing for larger meta-analyses (Adams et al., 2015; Wang et al., 2018). Although both methods have specific limitations regarding taxonomic resolution, limit of specificity, and artificial bias are important when analyzing different types of samples. An integrative approach using both techniques could be implemented to rapidly advance the field, although this requires higher experimental costs (Metcalf et al., 2017; Hillmann et al., 2018).

Importantly, results of microbiome studies vary due to differences in sampling, storage, processing, and data analysis (e.g., machine learning classification models) (Clarke et al., 2017; Kaszubinski et al., 2020a). Thus, while the field expands, forensically oriented studies should contain standardized protocols with validated techniques. These methods should ensure reproducibility, sensitivity, and quantitative accuracy while defining and delineating the limitations (e.g., expected error rates, limit of detection, and limit of specificity) (Clarke et al., 2017; Metcalf et al., 2017). Minimizing the distinct impact of these variables on microbial profiling to reduce bias and skewing of the detected microbial composition is crucial in forensic evidence.

## Future Perspectives and Concluding Remarks

Microbial forensics holds much potential; however, to integrate highly dimensional microbial data into routine investigative casework, several aspects need to be clarified. A key question is to what extent and for how long various individual factors shaping the vaginal, skin, and oral/salivary microbiome during life also play a role after death. These body sites are often inhabited by Gram-positive bacteria that are potentially more resistant to environmental and temporal degradation compared to Gram-negative bacteria and human DNA. In addition, vaginal, skin, and oral/saliva samples are routinely collected as critical components of sexual assault and femicide cases. Importantly, many sensitive individual characteristics can be associated with microbiome composition; however, the magnitude of these effects requires comprehensive investigation. A better understanding of the complex human body ecosystem during life and after death is necessary with the establishment of anthropological research facilities over different continents studying diverse populations and body sites. Hereby, we can facilitate discoveries especially related to female health and safety by comprehending how the post-mortem disturbance in the body homeostasis and its microbial communities make it more susceptible to the influences of the surrounding environment. While studies and regulations are complex specifically for the forensic field, the current and potential future possibilities of microbial forensics in phenotyping, identifying individuals, minimum PMI estimation, and the source of origin of a sample are highly important to consider and develop.

## Author Contributions

SA, IS, and SL conceived and designed the manuscript. SA wrote the manuscript. IS made the figure. SA, IS, RD, EJ, and SL critically reviewed the manuscript and contributed with special attention towards their specific expertise. All authors contributed to the article and approved the submitted version.

## Conflict of Interest

The authors declare that the research was conducted in the absence of any commercial or financial relationships that could be construed as a potential conflict of interest.

## Publisher’s Note

All claims expressed in this article are solely those of the authors and do not necessarily represent those of their affiliated organizations, or those of the publisher, the editors and the reviewers. Any product that may be evaluated in this article, or claim that may be made by its manufacturer, is not guaranteed or endorsed by the publisher.
